# Changes in Electrocardiogram in Patients With Spontaneous Subarachnoid Hemorrhage: A Cross-Sectional Study

**DOI:** 10.7759/cureus.40045

**Published:** 2023-06-06

**Authors:** Binod Poudel, Prasanna Karki, Samjhana Panta, Aastha Lamsal, Parash Koirala, Surya Devkota, Gopal Sedain, Mohan R Sharma

**Affiliations:** 1 Internal Medicine, Tribhuvan University Institute of Medicine, Kathmandu, NPL; 2 Intensive Care Unit, Tribhuvan University Institute of Medicine, Kathmandu, NPL; 3 Internal Medicine, Kist Medical College, Lalitpur, NPL; 4 Cardiology, National transplant centre, Kathmandu, NPL; 5 Cardiology, Manmohan Cardiothoracic Vascular and Transplant Center, Institute of Medicine, Tribhuvan University Teaching Hospital, Kathmandu, NPL; 6 Neurosurgery, Tribhuvan University Institute of Medicine, Kathmandu, NPL

**Keywords:** arrhythmia, emergency neurosurgery, prevalance studies, spontaneous subarachnoid hemorrhage, electrocardiogram

## Abstract

Background

Electrocardiographic (ECG) changes are frequently reported findings in patients with subarachnoid hemorrhage (SAH). We conducted a retrospective descriptive study to assess the prevalence of electrocardiographic changes in patients with non-traumatic SAH.

Methods

In this single-center retrospective cross-sectional study, ECG recordings of 45 patients who presented to Tribhuvan University Teaching Hospital in the year 2019 with SAH were collected and analyzed to detect any abnormalities.

Results

In our study, 88.8% of patients had some form of ECG abnormality. The most common ECG abnormalities associated with SAH were QTc prolongation, T-wave abnormalities, and bradycardia, which were found, respectively, in 35.5%, 24.4%, and 24.4% of the patients. Other ECG changes we observed include ST depression, large U-waves, atrial fibrillation, and premature ventricular contractions.

Conclusion

Morphological and rhythm abnormalities are frequently observed in patients with SAH, which may cause diagnostic dilemmas and unnecessary workups. Further studies are required to evaluate their significance and correlate ECG changes with clinical outcomes.

## Introduction

Intracranial hemorrhages (ICHs) are classified, depending on the cause, to be traumatic and non-traumatic, and the anatomical site further classifies ICHs into four main groups: epidural hemorrhage, subdural hemorrhage, subarachnoid hemorrhage (SAH), and intraparenchymal hemorrhage. SAH is defined as bleeding into the subarachnoid space, which is a potential space beneath the arachnoid membrane and above the pia mater. Subarachnoid hemorrhage is a worldwide health burden with high mortality and morbidity among survivors. The overall incidence of SAH is 7-11 per 100 000 inhabitants/year with a higher incidence in Japan and Finland [1]. Most SAHs are caused by ruptured saccular aneurysms. Other causes include trauma, arteriovenous malformations/fistulae, vasculitides, intracranial arterial dissections, amyloid angiopathy, bleeding diathesis, and illicit drug use. The aneurysmal rupture is most common in the age group of 50 to 55 years [2].

Acute stress on the nervous system has been known to affect the heart both structurally and functionally and is described in the literature as neurogenic stress cardiomyopathy (NSC). The occurrence of NSC is reported more frequently in some types of intracranial hemorrhage such as non-traumatic SAH than in others [3]. Despite tremendous advances in cardiac evaluations, an electrocardiograph (ECG) remains to be an essential tool to rule out cardiac illness, especially in emergency setups. The reported prevalence of ECG changes in patients with SAH ranges from 71% to 100% [3-5]. This might have major implications for the emergency management of the patient. Moreover, the presence of concomitant heart illness in this age group cannot be denied. 

ECG changes being that common, there have been both prospective and retrospective studies as well as holter monitoring of patients with SAH to find out its prevalence and significance. In previous large-sample studies, commonly detected abnormalities were large U wave, T wave abnormality, QTc prolongation, high R wave, ST depression, and bradycardia [4,5]. There have also been reports of cases where SAH has presented as a complete heart block and transient ST elevated myocardial infarction [6,7]. Some studies have tried to evaluate the significance of such ECG changes and there is evidence that the electrocardiographic abnormalities, especially abnormal Q or QS wave and nonspecific ST, T changes, may predict the development of neurogenic pulmonary edema within 24 hours in adult patients with spontaneous SAH [8]. This suggests a comprehensive knowledge of the ECG changes is pertinent in the management of SAH.

Evidence regarding the prevalence of ECG changes in SAH is lacking in our region. The aim of our study is to determine ECG changes in spontaneous SAH presenting over a period of one year in a tertiary hospital in Nepal.

## Materials and methods

Setting

This study was conducted at the Tribhuvan University Teaching Hospital, Kathmandu, Nepal, between Jan 2019 and Dec 2019. This hospital is the largest in Nepal, has a large patient flow, and has a department of neurosurgery. It was carefully selected for the review of such data.

Study design 

This retrospective observational study was approved by the Institutional Review Committee of Institute of Medicine, Tribhuvan University, with reference number: 501(6-11)E2. Structured proforma was used to record the data retrospectively for the admitted patients. We reviewed the records of patients admitted between January to December 2019.

Inclusion criteria

Patients above the age of 18 years with the diagnosis of subarachnoid hemorrhage who were admitted to the neurosurgery ward of the hospital were enrolled in the study. 

Exclusion criteria 

Patients were excluded if they had any of the followings: 1. Had a history of cardiovascular disease; 2. Was under antiarrhythmic treatment in the prior 6 months including beta blockers or any other drugs that could alter ECG recording; 3. Electrolyte abnormalities including severe hypokalemia (Serum K+ >6.0mEq/L), hyperkalemia (Serum K+ <3mEq/L), and hypocalcemia (Serum Ca <8mEq/L); 4. Diuretic treatment in the past month.

Study parameter

The diagnosis of subarachnoid hemorrhage was considered in any patient with severe and sudden onset or rapidly escalating headache and was confirmed by findings suggestive on computed tomography (CT) scan. Clinically suspicious but those negative on CT scan were subjected to lumbar puncture and CT angiography. We included only spontaneous cases of subarachnoid hemorrhage in our study. Demographic characteristics including age, sex, and pertinent medical history (diabetes mellitus, hypertension) of the patients were collected. We also collected whether SAH was aneurysmal or non-aneurysmal and whether it was repaired during the active stage. The neurological status of the patient including the Hunt-Hess score, World Federation of Neurological Surgeons (WFNS) score, reactivity, and size of the pupil was noted from the admission file. Table 1 summarizes the neurological grading scale used during our study, and both Hunt-Hess and WFNS scales are well standardized and their association with clinical outcomes is well described. 

**Table 1:** SAH clinical grading scales - Hunt-Hess and WFNS scoring WFNS: World Federation of Neurological Surgeons; GCS: Glasgow Coma Scale; SAH: subarachnoid hemorrhage [9,10]

World Federation of Neurological Surgeons Scale (WFNS)			Hunt and Hess Scale	
Grade	GCS	Neurologic Examination	Grade	Neurologic Examination
1	15	No motor deficit	1	Awake, alert, no cranial nerve or motor deficits, mild headache, minimal or no nuchal rigidity
2	13-14	No motor deficit	2	Awake, alert, moderate to severe headache, nuchal rigidity, no motor deficits,may have cranial nerve deficit
3	13-14	Motor deficit	3	Confusion or lethargy, with or without mild focal neurologic deficits
4	7-12	With or without motor deficit	4	Stuporous, more severe focal neurologic deficit
5	3-6	With or without motor deficit	5	Comatose, motor posturing or no motor response

ECG analysis 

A 12-lead Electrocardiography (ECG) was performed for all patients at the time of admission on a calibrated device with a paper speed of 25 mm/sec and a standard amplitude of 10 mm/mV. The ECG performed closest to the time of presentation was used in case of multiple ECG readings. All ECGs were interpreted by two cardiologists who were blinded to the clinical data of the patient. The following parameters were evaluated for each ECG:

1. Rhythm abnormalities 

1a. Heart rate (Bradycardia if HR <60 beats per minute (bpm) and Tachycardia if HR> 100 bpm) 

1b. Arrhythmias including Atrial fibrillation, Premature Ventricular Contractions (PVCs) 

2. Morphological ECG changes

2a. Poor progression of R waves

2b. ST Depression (>= 0.1 mV; 80 ms post J point)

2c. T-wave abnormalities (peaked T-waves, flat T-waves, and T-wave inversion) 

2d. Corrected QTc interval (calculated as QT interval divided by the square root of RR interval in seconds): considered prolonged if more than 440 in males and 460 in females. 

2e. Large U wave (amplitude >1mm)

2f. Axis deviation

Statistical analysis 

Data were compiled, edited, and checked as per a proforma to maintain consistency. The data was recorded and analyzed in Microsoft Excel for Windows (Microsoft Corp., Redmond, USA).

## Results

During the period of one year, a total of 52 adult patients with non-traumatic SAH were treated in this center, out of which only 45 were included in our study; the remaining seven were excluded because of coexisting electrolyte abnormalities. Demographic and clinical characteristics are summarized in Table 2. The mean age of patients was 54.3 ± 13.47 years. Of these patients, 30 (66.67%) were females. The average time of presentation was 44.35 ± 29.35 hours. Only two (4.4%) cases presented after a week while 39 (86.6%) cases presented within 72 hours. 12% of patients had some comorbidity - either diabetes or hypertension or both.

**Table 2:** Baseline characteristics of patients with SAH ACOM: Anterior Communicating Artery; PCOM: Posterior Communicating Artery; MCA: Middle Cerebral Artery; ICA: Internal Carotid Artery; WFNS: World Federation of Neurological Surgeons; SAH: Subarachnoid Hemorrhage

Clinical details				Number	Percentage	
Age				54.3±13.47		
Sex(F)				30	66.67	
Time of presentation				44.35±29.35		
Hypertension				12	26.66	
Diabetes				4	8.88	
Localization of hemorrhage						
ACOM				16	35.55	
PCOM				7	15.55	
MCA				8	17.77	
ICA				2	4.44	
Perimesencephalic				4	8.88	
Negative ct angiography				6	13.33	
Multiple				2	4.44	
Neurological status at presentation						
Hunt-Hess score						
Hunt Hess 1				1	2.22	
Hunt Hess 2				35	77.77	
Hunt Hess 3				9	20	
Hunt Hess 4				0	0	
WFNS						
WFNS 1				31	68.88	
WFNS 2				9	20	
WFNS 3				3	6.66	
WFNS 4				2	4.44	
Management						
Clipping				31	68.88	
Coiling				2	4.44	
Conservative				11	24.44	
Deferred treatment				1	2.22	
Death				4	8.88	

Characteristics of SAH

An initial CT scan localized the aneurysm in 39 (86.6%) patients out of which 16 (35.5%) localized in the anterior circulation. Six (13.3%) patients with spontaneous SAH were non-aneurysmal in origin. Most patients in our study had Hunt-Hess 2 and WFNS 1 scores at the time of presentation. Of these patients, 31 (68.8%) received clipping and 11 (24.2%) opted for conservative management. Only two patients underwent coiling. We should acknowledge the fact that coiling was not available at the study site and patients were referred to another center if they preferred coiling.

Characteristics of ECG abnormalities

The prevalence of different ECG changes is summarized in Table 3. Out of the 45 patients enrolled in the study, 40 (88.8%) patients had some form of ECG abnormality. Isolated rhythm abnormality was present in six (13.3%) patients, all presenting as bradycardia. All other 34 (75.5%) patients had concomitant rhythm and morphological changes in ECG.

**Table 3:** Prevalence of ECG abnormality

Abnormality			Number	Percent
Rhythm abnormality				
Mean heart rate			69.83±12.04	
Bradycardia			11	24.44
Atrial fibrillation			1	2.2
Sinus arrhythmia			1	2.2
Premature Ventricular contraction			1	2.2
Morphological abnormality				
Left Axis deviation			7	15.55
P mitrale			4	8.88
Poor progression of R wave			4	8.88
ST depression			4	8.88
Left Bundle Branch Block			1	2.22
T-wave abnormality				
Peaked T-wave			3	6.66
Flat T-wave			1	2.22
T- wave inversion			7	15.55
Corrected QT interval				
Prolonged			16	35.55
Shortened			2	4.44
U-wave			5	11.11

Rhythm abnormality

**Figure 1:**
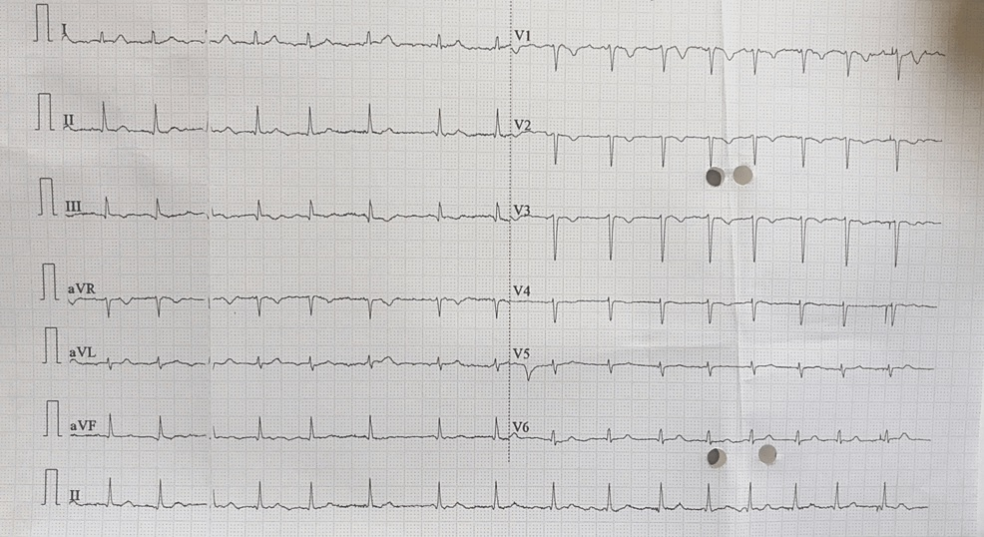
Representative image of rhythm abnormality with a 12 lead ECG of 75 years female with SAH The ECG reveals an irregular rhythm with absent p waves (atrial fibrillation) with T-wave inversion in V1 to V3. SAH: Subarachnoid hemorrhage

The heart rate was found to be 69.8 ± 12.04. Isolated rhythm abnormality was present in six (13.3%) patients, all presenting as bradycardia. Bradycardia was present in a total of 11 (24.4%) of patients. We encountered single cases of atrial fibrillation (Figure 1), sinus arrhythmia, and premature ventricular contraction. 

Morphological abnormality 

**Figure 2:**
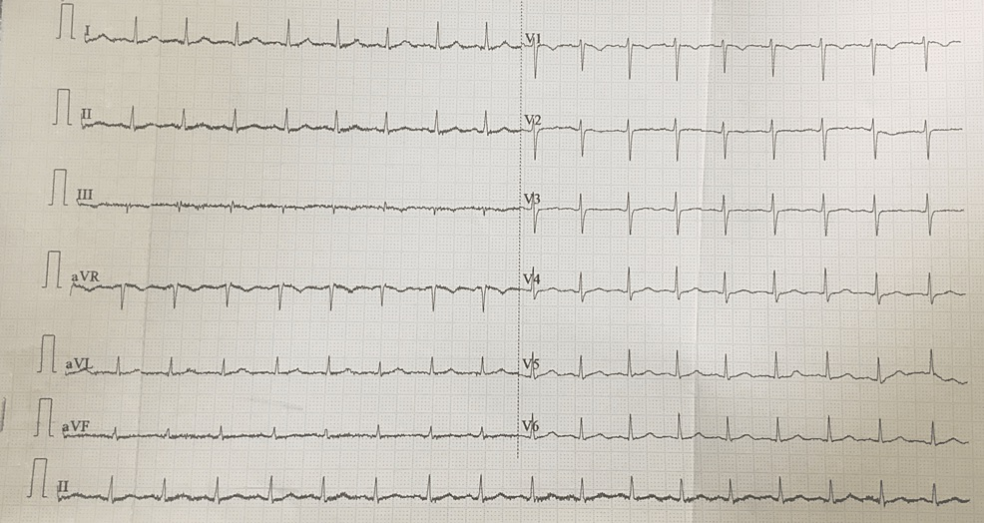
12-lead ECG of a 34-year-old female with SAH Recordings reveal prolonged QTc of 468 with nonspecific T- wave changes. SAH: Subarachnoid Hemorrhage

Out of the 34 patients with some form of morphological abnormality, a total of 12 (26.6%) patients had morphological changes without rhythm abnormality. The most frequent of these was abnormal QTc. Sixteen (35.5%) patients had prolonged QTc interval. Figure 2 shows a representative image showing the most commonly encountered abnormality, i.e., prolonged QTc.

T wave abnormality was also frequently encountered. A total of 11 (24.4%) patients had a T wave abnormality. Seven patients (15.5%) developed a T wave inversion.

Left axis deviation was present in seven (15.5%) cases. P mitrale was encountered in four (8.8%) and ST Depression in four (8.8%) cases. None of the patients had ST elevation. One patient presented as a left bundle branch block. U wave was detected in five (11.1%) patients.

## Discussion

The mechanism by which ECG changes occur in SAH is not clear. It has been suggested that SAH has been associated with raised plasma norepinephrine and autonomic sympathetic activity which is primarily responsible for most of the ECG abnormality [11]. There have been animal studies where cardiac ischemic changes and arrhythmias were induced by hypothalamic stimulation [12]. There is an increased incidence of raised intracranial pressure in patients with SAH and raised intracranial pressure can mediate ECG abnormalities [13,14]. Transient coronary vasospasm following SAH may also mediate ECG changes [15].

Prevalence

The reported prevalence of ECG changes in SAH ranges from 71 to 100% [3-5]. In our study, 88.8% of patients had some form of ECG abnormalities. Reported prevalence is higher in studies in which ECG recordings were retrieved within 48 hours or serial ECG recording was done [5]. In our study, the most common ECG abnormalities were QTc prolongation, T-wave abnormality, and bradycardia, found in 35.5%, 24.4%, and 24.4% of patients, respectively. We also observed left axis deviation, ST depression, and U wave in a minority of patients. 

In a previous study of 406 patients with SAH, Rudehill et al detected large U wave, T wave abnormality, QTc prolongation, high R wave, and ST depression in 47%, 32%, 24%, 19%, and 15%, respectively [4]. A similar study by Brouwers et al, who reviewed serial ECG prospectively in 61 patients, found T wave abnormality, bradycardia, ischemic ST changes, large U wave, and QTc prolongation in 59%, 50%, 50%, 44%, and 39%, respectively [5]. Compared to our study, Brouwers et al enrolled 77% of patients within 24 hours and remaining all patients within 72 hours and concluded that studies in which surveillance is started later in the course of illness may miss significant data. Excluding the two patients who presented to our center after weeks, 43 presented in 44.35 ± 29.35 hours. This delayed presentation may be attributable to fewer morphological changes than in the previous studies. 

QTc changes 

The most common ECG abnormality noted in our study is QTc prolongation, which is a measure of delayed ventricular repolarization. Patients with QTc prolongation are susceptible to arrhythmias including ventricular tachycardia and torsades de pointes [16]. QTc prolongation may be attributed to increased plasma catecholamines, corticosteroids, and potassium levels. In a multivariate analysis of risk factors for QT prolongation in 100 patients following SAH by Fukui et al., hypokalemia was found to be an independent risk factor for severe QTc prolongation. They recommended, a patient with SAH when hospitalized during the acute phase of SAH, as a rule, potassium-free intravenous fluids should not be used as initial fluid therapy, particularly in female patients. Avoidance of drugs that prolong ventricular repolarization (quinidine, procainamide, and disopyramide) is mandatory [17].

T wave changes 

Among the T wave abnormalities, T wave inversion was the most common finding in our study. Typical transient, symmetric, and deep inverted electrocardiogram T waves in the setting of stroke are commonly referred to as cerebral T waves. Myocardial infarction is an important cardiac cause of T wave inversion [18]. There have been case reports where patients with T wave inversion, ST segment elevation, and increased troponin I were later found to have SAH in the CT scan [19]. T wave abnormalities can provide added evidence to support the clinical diagnosis but T wave must be considered along with QRS and ST segment abnormalities. T wave inversion along with prolonged QT interval is more likely to be associated with cerebrovascular accidents while that with normal QTc interval is likely associated with myocardial infarction. Inverted T waves produced by myocardial ischemia are classically narrow and symmetric [20].

ST depression

We observed four patients who had ST depression and all of them had ST depression in three or more precordial leads. But we did not encounter any cases of ST elevations. There has been a case report where a patient has had an off-hospital cardiac arrest and had ST elevation with increased cardiac biomarker but normal coronary angiography and was later found to have SAH. In a study of 15 cases of electrocardiographic abnormalities suggestive of recent myocardial infarction or ischemia in association with SAH four patients with such abnormalities died and had a normal heart autopsy [21]. They concluded without a good history or clinical findings of heart disease, an electrocardiogram showing changes suggestive of recent infarction or acute myocardial ischemia should be interpreted as indicative of intracranial rather than of heart disease. A minority of our patients had left axis deviation and U wave but their prevalence is reportedly higher in other studies [22]. Also, their clinical significance and implication are minimal.

Rhythm abnormalities

Cardiac arrhythmias in SAH are thought to occur secondary to acute central nervous dysfunction and sudden increased intracranial pressure. Rhythm abnormalities were relatively rare than morphological abnormalities in our study. Sinus bradycardia was observed in 24.4% of our cases with one case of atrial fibrillation and one case of premature ventricular contractions. In a prospective study of 580 spontaneous SAH patients by Frontera et al cardiac arrhythmias occurred in 4.3% of patients with atrial fibrillation and flutter being the most common finding [23]. They concluded that arrhythmias were associated with an excess ICU stay of five days and an increased risk of death or severe disability. Di Pasquale et al detected cardiac arrhythmias in 90% of the patients by Holter monitoring and the frequency and severity were higher in subjects studied within 48 hours [24]. There have been case reports where SAH has been associated with life-threatening arrhythmias [25]. So, all patients with SAH must be kept under continuous cardiac rhythm monitoring in Neuro-ICU, especially in the first 48 hours for early detection of life-threatening arrhythmias and prevention of their complications. 

Implications

ECG changes, though common in SAH, are not an accurate predictor of myocardial dysfunction, rather myocardial dysfunction is related more closely to the severity of neurological dysfunction caused by SAH [26]. However, it should be acknowledged that ECG abnormalities, specifically corrected QT interval, are independently associated with an increased risk of neurogenic pulmonary edema and delayed cerebral ischemia, whereas ST depression is independently associated with an increased risk for in-hospital death [27]. The correct interpretation of ECG is required to prevent delays in treatment and wasteful investigations. Similarly, continuous cardiac rhythm monitoring of patients with SAH is essential for early detection and treatment of life-threatening cardiac arrhythmias. QT prolongation, a common finding in SAH, warrants avoidance of potassium-free fluid as initial fluid therapy and drugs prolonging ventricular repolarization. Proper knowledge about the pathophysiology of ECG changes in SAH patients may make heart donations possible in brain-dead patients of SAH with ECG abnormalities [22].

## Conclusions

This study demonstrates that both morphological and rhythm abnormalities are frequently encountered in patients with SAH. Eighty-eight percent of patients had some form of ECG abnormality. QTc prolongation was most commonly encountered. Though ST-T changes and U wave were among the most frequent abnormalities in past studies, we observed few such cases. We recommend further prospective studies with serial ECG or Holter recording in study populations like ours. 
